# Transmission risk of two chikungunya lineages by invasive mosquito vectors from Florida and the Dominican Republic

**DOI:** 10.1371/journal.pntd.0005724

**Published:** 2017-07-27

**Authors:** Barry W. Alto, Keenan Wiggins, Bradley Eastmond, Daniel Velez, L. Philip Lounibos, Cynthia C. Lord

**Affiliations:** Department of Entomology and Nematology, Florida Medical Entomology Laboratory, University of Florida, Vero Beach, Florida, United States of America; Institut Pasteur, FRANCE

## Abstract

Between 2014 and 2016 more than 3,800 imported human cases of chikungunya fever in Florida highlight the high risk for local transmission. To examine the potential for sustained local transmission of chikungunya virus (CHIKV) in Florida we tested whether local populations of *Aedes aegypti* and *Aedes albopictus* show differences in susceptibility to infection and transmission to two emergent lineages of CHIKV, Indian Ocean (IOC) and Asian genotypes (AC) in laboratory experiments. All examined populations of *Ae*. *aegypti* and *Ae*. *albopictus* mosquitoes displayed susceptibility to infection, rapid viral dissemination into the hemocoel, and transmission for both emergent lineages of CHIKV. *Aedes albopictus* had higher disseminated infection and transmission of IOC sooner after ingesting CHIKV infected blood than *Ae*. *aegypti*. *Aedes aegypti* had higher disseminated infection and transmission later during infection with AC than *Ae*. *albopictus*. Viral dissemination and transmission of AC declined during the extrinsic incubation period, suggesting that transmission risk declines with length of infection. Interestingly, the reduction in transmission of AC was less in *Ae*. *aegypti* than *Ae*. *albopictus*, suggesting that older *Ae*. *aegypti* females are relatively more competent vectors than similar aged *Ae*. *albopictus* females. *Aedes aegypti* originating from the Dominican Republic had viral dissemination and transmission rates for IOC and AC strains that were lower than for Florida vectors. We identified small-scale geographic variation in vector competence among *Ae*. *aegypti* and *Ae*. *albopictus* that may contribute to regional differences in risk of CHIKV transmission in Florida.

## Author summary

The emergence of mosquito-borne chikungunya virus in the Americas starting in 2013 has been associated with geographically widespread outbreaks of human illness. Transmission of chikungunya virus in the U.S. is a major public health risk, especially in Florida where the environmental conditions are favorable for the two main mosquitoes involved in transmission. We measured susceptibility to infection and transmission for Florida *Aedes aegypti* and *Aedes albopictus* mosquitoes for two emergent strains of chikungunya virus (Indian Ocean and Asian strains). Both mosquito species showed high susceptibility to infection and rapid spread of the virus throughout the body of the mosquito, including the saliva for both emergent strains of chikungunya virus. *Aedes albopictus* had higher body infection and transmission of the Indian Ocean strain sooner after feeding on chikungunya virus infected blood than *Ae*. *aegypti*. *Aedes aegypti* had higher body infection and saliva infection later during infection with the Asian strain of chikungunya virus than *Ae*. *albopictus*. We also observed declines in body infection and transmission over time, suggesting that transmission risk declines with length of infection. The information here will be useful as parameters in models of risk of chikungunya virus transmission.

## Introduction

Native to Africa, chikungunya virus (CHIKV) emerged to produce intermittent outbreaks from the 1950s in Southeast Asia (Asian CHIKV lineage) and regional outbreaks in India in the 1960s and 1970s [1, 2]. Chikungunya also emerged in Kenya in 2004 (Eastern/Central/Southern African, ECSA, CHIKV lineage), followed by an outbreak of chikungunya fever on the island of La Réunion in 2005–2006 involving the Indian Ocean CHIKV (IOC) lineage, a descendent of the ECSA CHIKV lineage [2, 3]. In 2013 an Asian lineage of CHIKV (AC) was detected and transmitted locally on St. Martin Island, a French collectivity in the Caribbean [4, 5], followed by spread throughout much of the Americas by 2015 [6]. The Old-World outbreaks of CHIKV in Kenya in 2004 [3] and islands of the Indian Ocean in 2005 subsequently spread to India and Europe including Italy and France [7, 8] involving more than one million cases. The outbreaks in Europe were one of the first demonstrations that CHIKV could extend its tropical/subtropical distribution into temperate regions using the Asian tiger mosquito vector *Aedes albopictus* (Skuse). Due to its ability to tolerate lower temperatures [9] *Ae*. *albopictus* occurs at more northern latitudes than *Aedes aegypti* (L.), which is usually considered the primary vector of CHIKV. The Old-World outbreaks were caused by the Indian Ocean strain of CHIKV [5, 10]. Asia is part of the invasive range of CHIKV where *Ae*. *aegypti* is the primary vector [11, 12]. The virus is native and endemic to Africa, where arboreal mosquitoes are part of its sylvan cycle, including members of the *Ae*. *furcifer-taylori* group [11, 13].

Chikungunya virus can cause widespread epidemics with infection rates exceeding 25% in some locations (e.g., La Réunion, Americas) [6, 14]. It is estimated that more than 4 million cases have occurred worldwide in the past 12 years [12]. Human CHIKV infection causes high fever, rash, headache, joint swelling, and joint pain [15]. Additionally, chronic musculoskeletal diseases may last for months to years following infection [16]. The widespread, invasive mosquito *Ae*. *albopictus* was the vector of CHIKV on La Réunion during 2005–2006 [17, 18] and likely the primary vector of an outbreak in 2007 in Gabon [19, 20]. A single mutation in the E1 protein of CHIKV enhanced infection and transmission in *Ae*. *albopictus* [21], a species that was considered to be secondary in importance to the primary vector *Ae*. *aegypti*. There is now evidence showing that there have been multiple independent events of CHIKV exposure to *Ae*. *albopictus* populations followed by development of this adaptive mutation [21]. This observation suggests the potential for outbreaks involving *Ae*. *albopictus* as the vector in regions where *Ae*. *aegypti* is rare or absent. The expansion of CHIKV in the Americas that began in 2013 increases the burden of disease in a region of the world recently invaded by Zika virus and where dengue is endemic. Local transmission in the U.S. is a major public health risk especially in Texas and Florida where both potential mosquito vector species reside, the environmental conditions promote vector abundance throughout much of the year, and there is a high potential for virus introduction [22]. The first documentation of locally-acquired cases of CHIKV in the continental U.S. states occurred in 2014 involving 11 cases in Florida [23].

During 2014, the Dominican Republic was one of the countries with the most numerous suspected CHIKV cases in the Americas [6, 24]. Although it remains unclear what accounts for the observed high number of cases, this country is highly urbanized and many people live in densely populated marginal barrios. These environmental conditions together with domestic storage of water and irregular trash collections foster conditions favorable for the proliferation of *Ae*. *aegypti* which is present in most major cities in the Caribbean Basin. Similar environmental conditions likely exist in other parts of the Americas that experienced a high number of CHIKV cases. Additionally, the Dominican Republic is a top destination for tourism, which may have facilitated introduction of CHIKV and subsequent local transmission. Variation in vector competence and the extrinsic incubation period is another possible explanation; *Ae*. *aegypti* originating from the Dominican Republic may be highly competent for CHIKV, and the high infection rates in humans are a product of an efficient vector population.

Studies to date have demonstrated distinct differences in vector competence of *Ae*. *aegypti* and *Ae*. *albopictus* depending on geographic origin of the mosquitoes and CHIKV lineage [25, 26]. *Aedes aegypti* tends to be a more efficient transmitter of the Asian and ancestral East/Central/South Africa (ECSA) lineages, whereas *Ae*. *albopictus* is a more competent vector of the Indian Ocean strain of CHIKV [21, 25, 27]. Although a few studies have assessed infection and viral dissemination in *Ae*. *aegypti* and *Ae*. *albopictus* from Florida [28–31], little is known about the ability of these *Aedes* to transmit CHIKV (i.e., transmission efficiency). [30] showed that 0–20% of *Ae*. *aegypti* and 7–21% of *Ae*. *albopictus* were capable of transmitting the Indian Ocean strain of CHIKV (LR2006-OPY1). However, that study used Florida-derived *Ae*. *aegypti* and *Ae*. *albopictus* that had been maintained as laboratory colonies for > 50 generations and may not be representative of field populations. Furthermore, the vector competences of *Ae*. *aegypti* and *Ae*. *albopictus* populations differ among the three CHIKV lineages (ECSA, West Africa, Asian) [25, 27], and transmission of Caribbean CHIKV from the Asian lineage is likely to be affected by genetic differences in vector competence of Florida *Aedes* vectors.

An assessment of the vector competence of 35 populations of American *Ae*. *aegypti* and *Ae*. *albopictus* for two strains (i.e., individual isolates) in the ECSA lineage and one strain in the Asian lineage of CHIKV revealed that viral dissemination was high for all mosquito populations [32]. However, transmission rates differed vastly among American populations (11–97%) suggesting that salivary gland infection/escape barriers affect vector competence among these two-vector species and their potential to transmit CHIKV. For the Indian Ocean and ancestral ECSA genotypes (both in the ECSA lineage) transmission efficiencies among F1 generation *Ae*. *aegypti* and *Ae*. *albopictus* from Vero Beach, FL were lower (<30%) than most other American populations of these species. However, the vector competence of these *Aedes* mosquitoes from Vero Beach was not tested for the Asian lineage of CHIKV. [32] provided preliminary information that Florida *Aedes* may differ from other American populations of these species in their response to CHIKV attributable to differences in salivary gland infection/escape barriers.

Little is known about the vector competence of *Aedes* mosquitoes for the Asian lineage of CHIKV responsible for the outbreak in the Americas, including Florida. Chikungunya virus in the Americas belongs to the Asian lineage, suggesting that *Ae*. *albopictus* will transmit at a lower rate than *Ae*. *aegypti* due to an adaptive constraint [33]. However, other studies have suggested that *Ae*. *albopictus* were as competent as *Ae*. *aegypti* for transmission of an isolate of CHIKV from Saint Martin Island belonging to the Asian lineage [34]. With the potential for a major CHIKV epidemic in Florida, there is a need to appraise the relative risk and emergence of Chikungunya fever in Florida. An assessment using meteorological driven models to inform baseline risk for local Zika virus transmission in the U.S., and presumably other viruses including CHIKV and dengue viruses, showed that cities in southern Florida and south Texas were highly suitable for *Ae*. *aegypti* and imported viral cases [22].

In this paper, we examine CHIKV disseminated infection and transmission in Florida mosquitoes for two putative vector species, *Ae*. *aegypti* and *Ae*. *albopictus*. We tested whether local populations of *Ae*. *aegypti* and *Ae*. *albopictus* show regional differences in susceptibility to infection and transmission to two emergent lineages of CHIKV, Indian Ocean (IOC) and Asian genotypes (British Virgin Islands, AC). As a baseline comparison, we compare susceptibility to infection and transmission *Aedes* vectors from Florida to *Ae*. *aegypti* from the Dominican Republic, one of the countries associated with the most numerous cases of CHIKV during the American outbreak in 2014. Although *Ae*. *albopictus* is present in the Dominican Republic, it is often found at far lower abundances than *Ae*. *aegypti* and so we focused on the latter species [35].

## Materials and methods

### Mosquitoes and viruses

**Fig 1:**
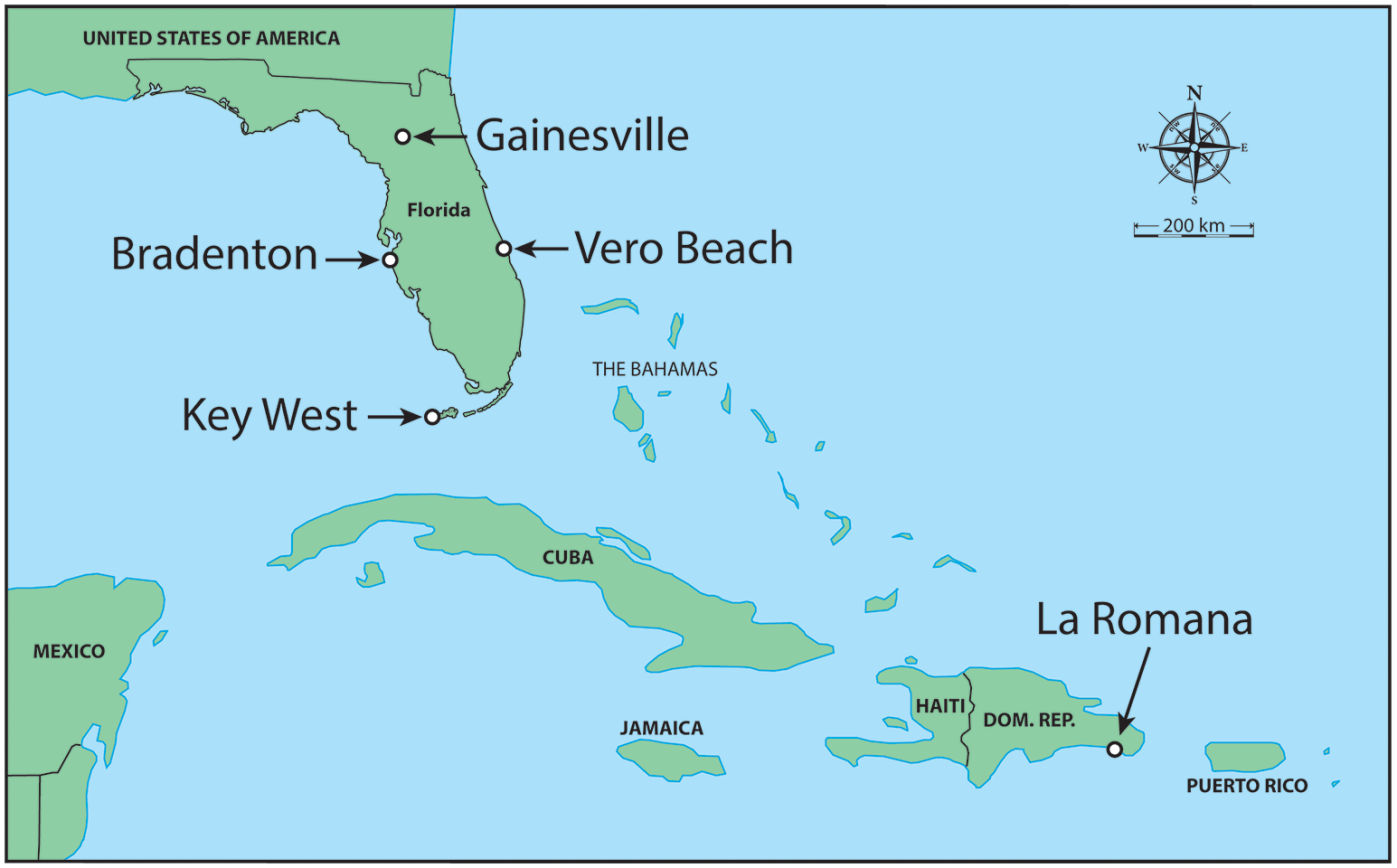
Collection sites of *Aedes aegypti* and *Ae*. *albopictus* across Florida and *Ae*. *aegypti* from the Dominican Republic.

We chose collection sites (Fig 1) based on distributions of these *Aedes* species across the state of Florida and areas where local arbovirus transmission (chikungunya, dengue, and Zika viruses) has been detected in areas where these vector species are present. Larval *Ae*. *aegypti* and *Ae*. *albopictus* were collected in 2014 from cemeteries or tire/salvage yards across Florida where these species are present alone or coexist [36]. Collection sites for *Ae*. *aegypti* included Manatee (Bradenton), Monroe (Key West), and Indian River/St. Lucie (Vero Beach or White City) Counties. We initially made collections from separate sites in Indian River Co. and St. Lucie Co. but later decided to combine these to augment sample size given their proximity to one another (23 km between sites). Collection sites for *Ae*. *albopictus* included Alachua (Gainesville), Manatee (Bradenton), and Indian River/St. Lucie (Vero Beach or White City) Counties. So, we included collections from distinct regions of Florida (East, West, North, and South) for our assessment of regional differences in susceptibility to infection and transmission of two emergent lineages of chikungunya virus among *Ae*. *aegypti* and *Ae*. *albopictus*. We also included a laboratory colony of *Ae*. *aegypti* originally collected in Orlando, FL (Orange Co.) and maintained in colony since 1952. Although no strong geographic genetic differentiation among Florida populations of *Ae*. *aegypti* has been reported, there is some evidence of genetic isolation of Florida Keys *Ae*. *aegypti* from mainland Florida [37]. We were provided with eggs of *Ae*. *aegypti* collected in 2014 from La Romana, Dominican Republic by the University of Texas Medical Branch which were propagated at the Florida Medial Entomology Laboratory for the CHIKV infection study. The inclusion of the Dominican Republic strain of *Ae*. *aegypti* enabled us to compare Florida *Aedes* vectors to a separate vector population involved in outbreaks with the most numerous cases of CHIKV in the Americas in 2014 [6].

Field-collected mosquitoes were reared to adulthood on a diet of equal parts of brewer’s yeast and liver powder larval food at 26–28°C. Pupae were collected daily and placed in vials with a cotton seal and upon emergence identified to species. Adults were provided with 10% sucrose solution and allowed to feed through hog casing membranes on commercially purchased defibrinated bovine blood (Hemostat Laboratories, Dixon, CA) once per week to propagate eggs. Larvae were reared at an approximate density of 150 larvae/L water in plastic photo trays (25cm width, 30cm length, 5cm height; Richard MFG Co. Fernandina Beach, FL, U.S.A) with 900 mL of water and 0.4 g larval food at hatching and supplemented again with the same amount 3–4 days later. Larvae developed to the pupal stage between 5–7 days after egg hatch. Adult males and females were held together for nine days in a cage (0.3^3^ m) in a climate controlled room (26–28°C, photoperiod of 14:10 light:dark) and provided with 10% sucrose solution. Females were placed in cylindrical cages (height x diameter: 10 cm by 10 cm, 50 females/cage) with mesh screening one day before exposure to CHIKV infected blood and deprived of sucrose but not water. The F_1-3_ generation progeny of field-collected *Ae*. *aegypti* and *Ae*. *albopictus*, including *Ae*. *aegypti* from the Dominican Republic, were used for the CHIKV infection studies in the biosafety level-3 virology facility at the FMEL in Vero Beach, FL.

### Viral isolates and propagation

A strain of CHIKV from the British Virgin Islands (BVI) (Asian lineage, GenBank accession: KJ451624), which is responsible for outbreaks that began in St. Martin in 2013, was obtained in December 2013 from an infected human. The Indian Ocean genotype (IOC) of CHIKV (LR2006-OPY1, GenBank accession: KT449801), responsible for the outbreak in the Indian Ocean region and parts of Europe [2], was isolated from a febrile patient in France who had been infected in La Réunion [38]. The virus isolates were obtained from the Centers for Disease Control and Prevention and the University of Texas Medical Branch in Galveston, TX. These CHIKV strains (passaged twice) were propagated in culture using African green monkey (Vero) cells, in which viral titer was determined by plaque assay [28].

### Mosquito infection

Ten to thirteen-day old adult females were provided with CHIKV infected defibrinated bovine blood (Hemostat, Dixon, CA) using an artificial membrane feeding system (Hemotek, Lancashire, United Kingdom) as described previously [39]. Aliquots of blood were stored at -80°C for later determination of virus titer. Briefly, to prepare fresh virus for mosquito infection, monolayers of Vero cells in T-175 cm^2^ flasks were inoculated with 500 μl of diluted stock CHIKV (multiplicity of infection, 0.1) and incubated for 1 hr at 37°C and 5% CO_2_ atmosphere, after which 24 mL media (M199 medium supplemented with 10% fetal bovine serum, penicillin/streptomycin and mycostatin) were added to each flask and incubated for an additional 47-hours. Mosquitoes were fed either a low dose (5.8 log_10_ pfu/ml) or high dose of CHIKV infected blood (8 log_10_ pfu/ml). After the feeding trials, fully engorged females were held in cylindrical cages and maintained at a 14:10 hour light:dark photoperiod and 30°C. To assess ability to transmit CHIKV mosquitoes were transferred to 37-mL plastic tubes (height x diameter: 8 by 3 cm) along with an oviposition substrate. Each tube held one mosquito and was fitted with a removable screen lid. Mosquitoes were deprived of sucrose for one day before the transmission trial started. Only *Ae*. *aegypti* from St. Lucie Co. were fed the low dose of the BVIC strain of CHIKV and held at 25°C or 30°C and tested for infection and salivary infection six days after ingesting CHIKV infected blood.

### Mosquito transmission and virus detection by qRT-PCR

Cohorts of mosquitoes were tested for transmission of CHIKV at 2, 5–6, and 12–13 days after feeding on a high dose of infected blood. Each tube containing a mosquito was presented with a honey-soaked filter paper (≈1 cm diameter) fastened to the inside of the lid. The honey was dyed with blue food coloring (McCormick) which provided a visual marker indicating that a mosquito fed on the honey and presumably deposited saliva during feeding. A similar system using FTA^®^ cards (Flinders Technology Associates filter paper) instead of filter paper has been used successfully as a surveillance system to detect arboviruses that exploits the fact that female mosquitoes expectorate virus in their saliva during feeding on sugar sources [40]. An initial assessment using FTA cards to test for ability to transmit CHIKV suggested a toxic effect on mosquitoes (early death) and so we switched to using filter paper as the substrate to collect mosquito saliva. Here we use this methodology as a proxy for potential to transmit CHIKV. Mosquitoes were examined with a flashlight for blue coloring in their crop after 24 and 48-hours during the transmission assay. Mosquitoes and filter paper were collected upon first detection of blue in the crop and frozen at -80°C and later analyzed for expectorated virus using quantitative (q) RT-PCR [28], so that CHIKV remained on the filter paper no longer than 24-hours before being frozen. Mosquitoes that did not feed on blue honey were not tested for CHIKV transmission. Cohorts of mosquitoes were tested for transmission of CHIKV at 2, 5–6, and 12–13 days after feeding on infected blood. Additionally, saliva was collected from these same mosquitoes in capillary tubes with immersion oil as described previously [41] after they had fed on blue honey for the second and third time points only. Additional studies (Alto et al., in preparation) indicate that the blue honey method is equivalent or slightly underestimates virus in saliva compared to capillary tube methods. All mosquitoes were immediately killed and stored at -80°C upon completion of each transmission assay. Mosquitoes were individually dissected and the bodies and legs were tested separately for the presence of CHIKV RNA by qRT-PCR using methods of [28]. Primers were designed to target a nonstructural polyprotein gene common to both lineages (accession ID of transcript, KU365292.1) with the following sequences: forward, 5'-GTACGGAAGGTAAACTGGTATGG-3': reverse, 5'-TCCACCTCCCACTCCTTAAT-3'. The probe sequence was: 5'-/56-FAM/TGCAGAACCCACCGAAAGGAAACT/3BHQ_1/-3' (Integrated DNA Technologies, Coralville, IA). Disseminated infection was calculated as the percent of infected legs from the total number engorged with blood. Transmission was calculated as the percent of saliva infected mosquitoes from the total number of mosquitoes with infected legs.

The legs and filter paper were homogenized separately in 1.0 mL of 199 media. The saliva from mosquitoes collected in capillary tubes was combined with 300 μL of media. A 140 μL sample of mosquito legs, filter paper, and saliva homogenate was used for RNA isolation using the QIAamp viral RNA mini kit (Qiagen, Valencia, CA) and eluted in 50 μL of buffer per the manufacturer’s protocol. CHIKV RNA was detected using the Superscript III One-Step qRT-PCR with Platinum Taq kit by Invitrogen (Invitrogen, Carlsbad, CA) as described previously [28]. Quantitative RT-PCR was performed with the CFX96 Real-Time PCR Detection System (Bio-Rad Laboratories, Hercules, CA) using primers and probes specific to the Asian and Indian Ocean lineages of CHIKV. The program for qRT-PCR was as follows; 50°C for 30 minutes, 94°C for 2 minutes, 39 cycles at 94°C for 10 seconds and 60°C for 1 minute, and lastly 50°C for 30 seconds. A standard curve method was used to express the titer of CHIKV of mosquito samples by comparing cDNA synthesis for a range of serial dilutions of CHIKV in parallel with plaque assays of the same dilutions of virus, expressed as plaque forming unit equivalents (pfue)/ml [42].

### Statistical analyses

Mosquito species, time, location, and species by time interaction effects on transmission were analyzed using maximum likelihood categorical analyses of contingency tables (PROC CATMOD, SAS 2002) based on the number of mosquitoes categorized for the presence or absence of CHIKV on the filter paper (first time point) and in capillary tubes (second and third time points). When significant treatment effects were found, follow-up analyses included pairwise comparisons of treatments, correcting for multiple comparisons using the sequential Bonferroni method. We chose this analysis for consistency and improved comparison to other CHIKV studies [28, 32, 34, 43, 44]. Separate analyses of transmission were performed for the first time point and combined for the second and third time points because different methods were used to collect saliva. Also, separate analyses were performed for each of the CHIKV lineages since these experiments were performed at different times. Maximum likelihood categorical analyses of contingency tables were used to test for treatment effects (see salivary infection methods) on viral dissemination efficiencies to gauge barriers to transmission (midgut escape barrier). Each infection experiment with *Ae*. *aegypti* and *Ae*. *albopictus* and CHIKV was conducted only once. Individual mosquitoes are the unit of replication and we analyzed infection responses by analysis of frequency distribution [45]. Analysis of variance was used to test for differences in virus titers in the legs and saliva of the individual mosquitoes. Significant effects were followed by Tukey-Kramer multiple comparisons among treatment least-squares means for pairwise comparisons.

## Results

Virus titer equivalents of infected blood were 5.8 log_10_ pfue/ml for the experiment exposing *Ae*. *aegypti* to a low dose of AC infected blood. *Aedes aegypti* and *Ae*. *albopictus* were exposed to approximately 8 log_10_ pfue/ml for the experiment with a high dose of CHIKV infected blood (September 9, 2015, IOC 8.1±0.1 log_10_ pfue/ml, AC 8.4±0.5 log_10_ pfue/ml (t-test, *p* = 0.08); September 10, 2015, IOC 8.2±0.1 log_10_ pfue/ml, AC 8.3±0.2 log_10_ pfue/ml (t-test, *p* = 0.39)). T-tests were performed between CHIKV lineages. The populations and mosquito species were distributed approximately evenly across the two feeding dates. Replication comes from blood sampled 5 to 14 times across feeding apparatuses for each feeding trial. These titers approximate the viral load in patients with symptomatic CHIKV infection [46].

### Infection and transmission in mosquitoes fed low titer blood

Preliminary studies using *Ae*. *aegypti* mosquitoes from St. Lucie Co. and AC identified baseline infection and disseminated infection rates at constant 25°C and 30°C six days after ingesting CHIKV infected blood. Although not significant (χ^2^ = 2.55, df = 1, *p* = 0.11), we observed 3-fold differences in susceptibility to infection between 30°C (10.5%, 67 mosquitoes tested) and 25°C (3.6%, 83 mosquitoes tested). Viral dissemination (χ^2^ = 0.05, df = 1, *p* = 0.83, 8 mosquitoes) and transmission (χ^2^ = 0.44, df = 1, *p* = 0.51, 8 mosquitoes) did not differ between the two temperatures. Viral dissemination rates of 100% and 40% were observed at 25°C and 30°C, respectively. Transmission of 33.3% and 0% was observed at 25°C and 30°C, respectively, using only the capillary tube method of collection of saliva.

### Infection and transmission in mosquitoes fed high titer blood

**Fig 2:**
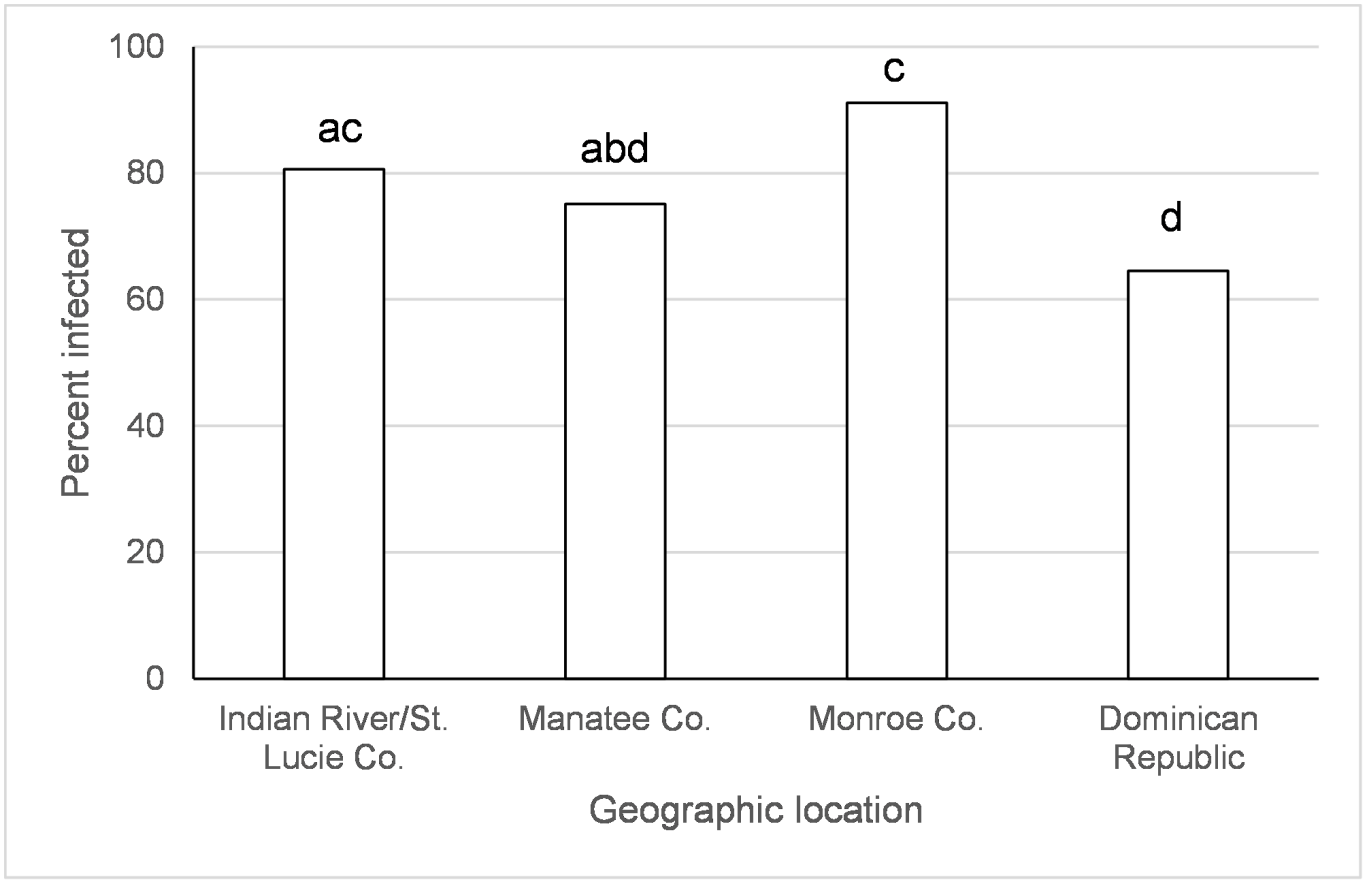
Disseminated infection of the Indian Ocean lineage of chikungunya virus in *Ae*. *aegypti*. Values associated with different letters show significant differences after correcting for multiple comparisons using the sequential Bonferroni method. Values average over the time treatment factor.

There was an effect of species, origin of *Ae*. *aegypti* population, and the species x time since exposure interaction on disseminated infections of IOC (Table 1). There were significantly more *Ae*. *albopictus* with disseminated infections at days 2 and 12, but not day 5, after IOC exposure than *Ae*. *aegypti* (day 2 *albo* (92.3%) *> aeg* (73.3%), χ^2^ = 13.6, df = 1, *p* = 0.0002; day 5 *albo* (88.0%) = *aeg* (89.5%), χ^2^ = 0.11, df = 1, *p* = 0.73; day 12 *albo* (94.7%) *> aeg* (75.6%), χ^2^ = 7.9, df = 1, *p* = 0.0049; Percentages combine over geographic populations in Table 2). *Aedes aegypti* from the Dominican Republic and Manatee Co., FL had lower or similar virus dissemination than *Ae*. *aegypti* from other locations in Florida (Fig 2).

**Table 1:** Treatment effects on the Indian Ocean lineage of chikungunya virus disseminated infection (leg infection) and transmission. *Aedes aegypti* = *aeg* and *Ae*. *albopictus* = *albo*.

Disseminated infection		
**Factor**	***χ***^***2***^	*p*
**Species**	4.6	0.03
**Location**	*aeg* = 29.8 *albo* = 0.52	*aeg*<0.0001 *albo* = 0.77
**Time**	0.94	0.62
**Species x Time**	6.3	0.04
**Transmission**		
**Factor**		
**Day 2**	***χ***^***2***^	*p*
**Species**	20.79	<0.0001
**Location**	*aeg* = 19.9 *albo* = 33.6	*aeg* = 0.0005 *albo*<0.0001
**Days 5 and 12**	***χ***^***2***^	*p*
**Species**	3.34	0.06
**Location**	*aeg* = 0.66 *albo* = 11.6	*aeg* = 0.88 *albo* = 0.003
**Time**	2.83	0.09
**Species x Time**	0.14	0.71

**Table 2:** Indian Ocean lineage of chikungunya virus disseminated infection and transmission for *Aedes aegypti* and *Ae*. *albopictus* from different geographic regions of Florida and the Dominican Republic. *Aedes aegypti* = *aeg* and *Ae*. *albopictus* = *albo*.

Geographic region^a^	Mosquito species	Day post infection	% disseminated infection (no. of mosquitoes)^b^	% transmission (no. of mosquitoes)^c^
**Indian River/St. Lucie Co.**	*aeg*	2	91.9 (37)	11.4 (35)
		5	93.0 (71)	40.9 (66)
		12	57.1 (28)	45.8 (24)
	*albo*	2	95.8 (48)	72.3 (47)
		5	60.0 (5)	66.6 (3)
		12	96.7 (31)	48.1 (27)
**Monroe Co.**	*aeg*	2	84.4 (90)	39.8 (83)
		5	100 (20)	22.2 (18)
		12	88.8 (54)	50.0 (50)
**Manatee Co.**	*aeg*	2	67.6 (71)	6.9 (58)
		5	80.9 (68)	46.0 (63)
		12	76.7 (60)	31.4 (51)
	*albo*	2	90.0 (30)	10 (30)
		5	100 (3)	100 (3)
		12	87.5 (8)	28.6 (7)
**Alachua Co.**	*albo*	2	88.5 (26)	8.3 (24)
		5	94.1 (17)	19.2 (26)
		12	94.4 (18)	5.5 (18)
**Dominican Republic**	*aeg*	2	37.1 (35)	5.9 (17)
		5	95.5 (22)	21.1 (19)
		12	61.1 (18)	53.3 (15)
**Laboratory colony (Orlando)**	*aeg*	2	ND	ND
		5	ND	ND
		12	ND	ND

^a^Geographic region of mosquito populations collected in Florida and the Dominican Republic.

^b^Disseminated infection corresponds to the percent of infected legs from the total number engorged with blood. A total of 760 mosquitoes were tested.

^c^Transmission corresponds to the percent of infected saliva mosquitoes from the total number of mosquitoes with infected legs. A total of 684 mosquitoes were tested.

ND, not determined.

**Fig 3:**
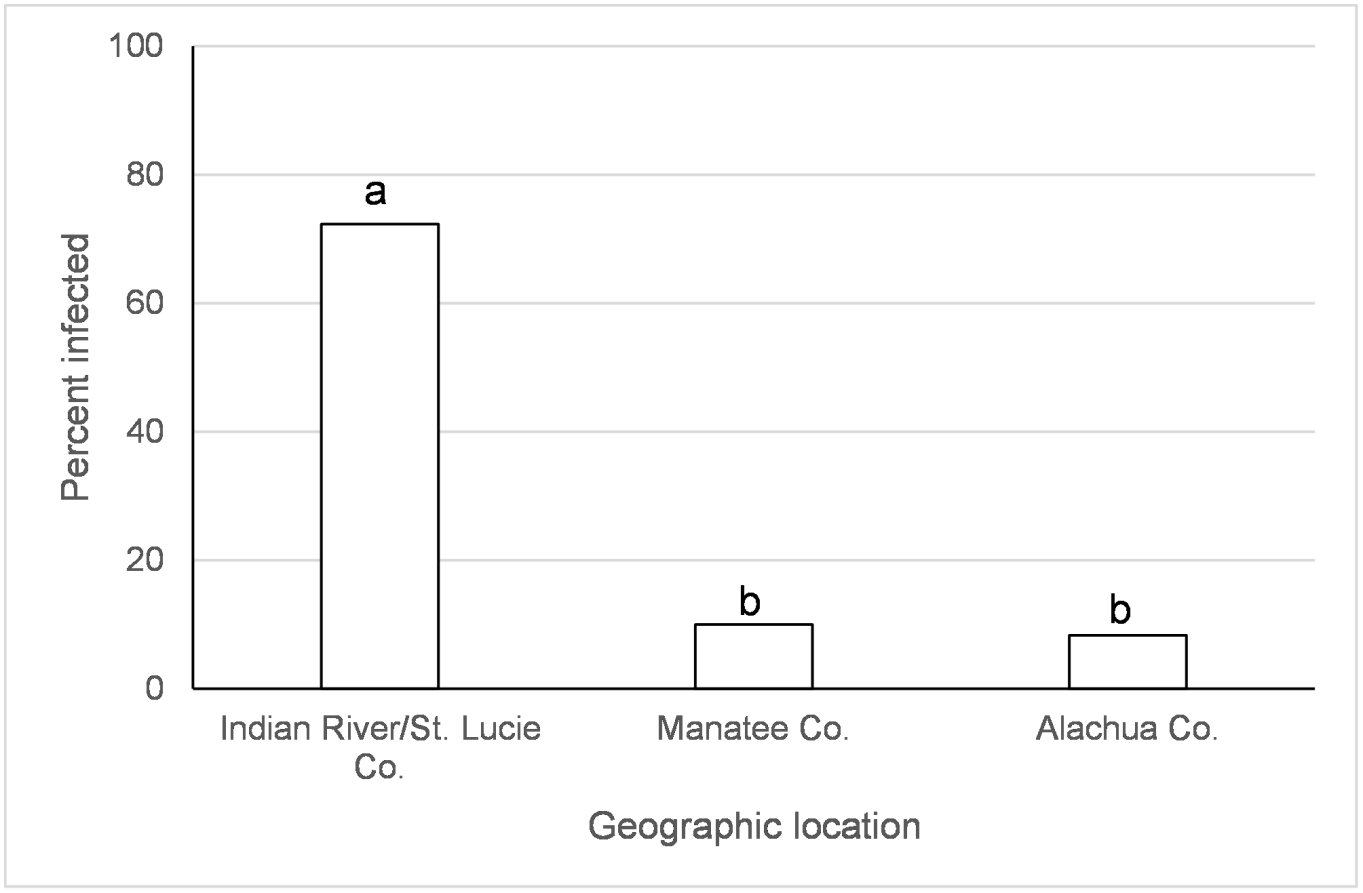
Transmission (day 2) of the Indian Ocean lineage of chikungunya virus in *Ae*. *albopictus*. Values associated with different letters show significant differences after correcting for multiple comparisons using the sequential Bonferroni method. Values average over the time treatment factor.

**Fig 4:**
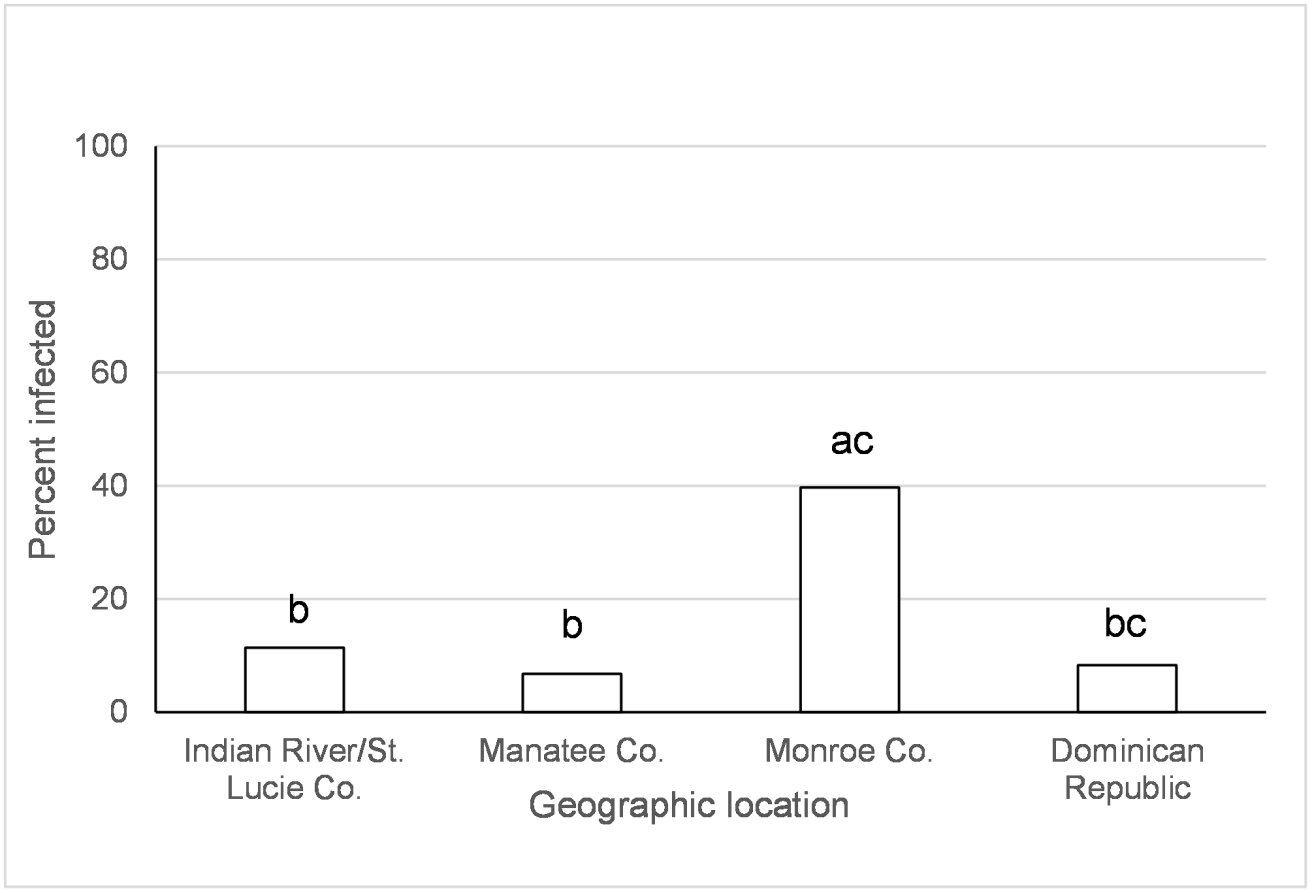
Transmission (day 2) of the Indian Ocean lineage of chikungunya virus in *Ae*. *aegypti*. Values associated with different letters show significant differences after correcting for multiple comparisons using the sequential Bonferroni method. Values average over the time treatment factor.

**Fig 5:**
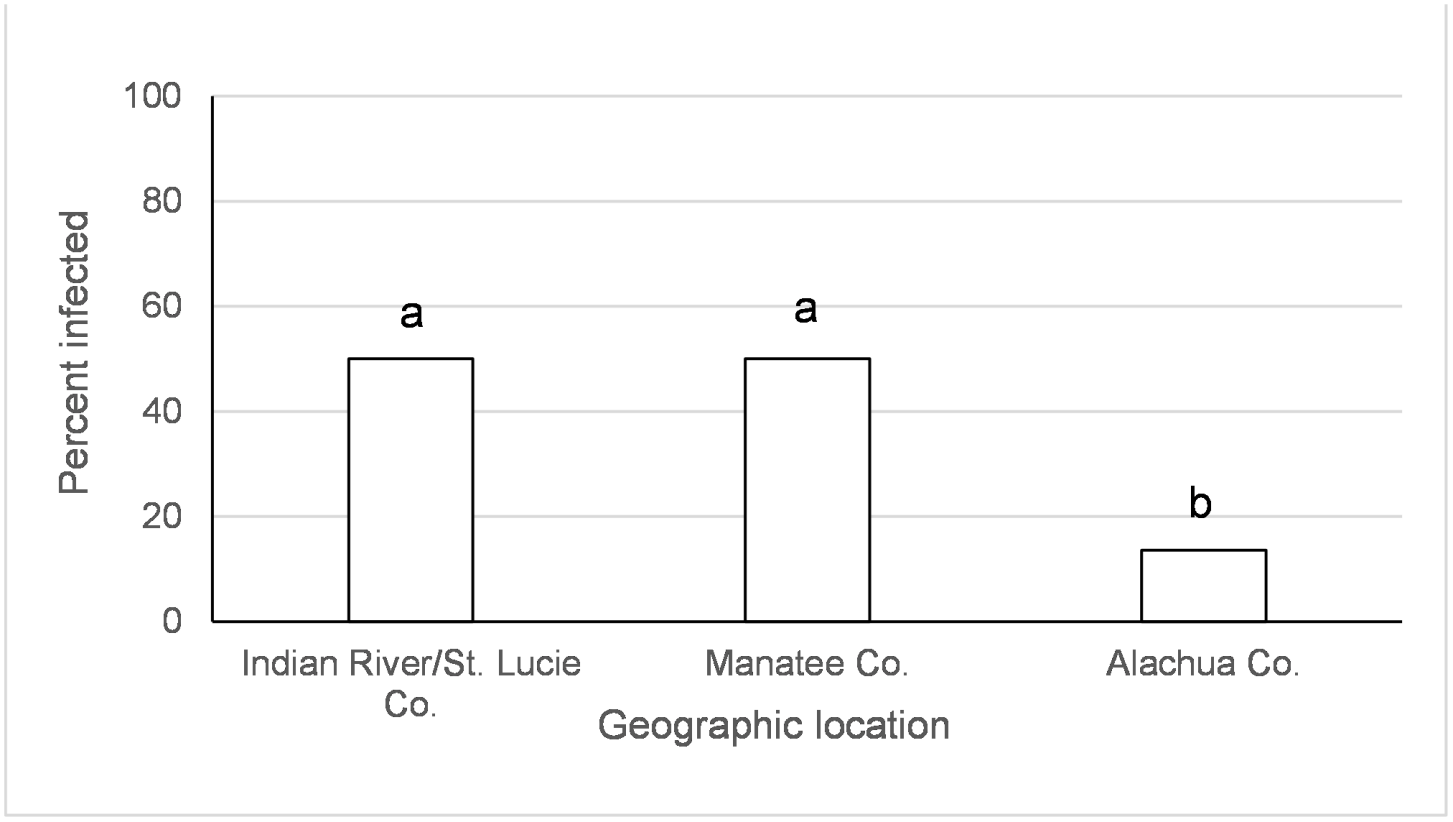
Transmission (days 5 and 12) of the Indian Ocean lineage of chikungunya virus in *Ae*. *albopictus*. Values associated with different letters show significant differences after correcting for multiple comparisons using the sequential Bonferroni method. Values average over the time treatment factor.

There was an effect of species, origin of *Ae*. *albopictus* population, and origin of *Ae*. *aegypti* on transmission of IOC two days following ingestion of infected blood (Table 1). *Aedes albopictus* transmission was higher than *Ae*. *aegypti* on day 2 after IOC exposure (*albo* = 38.6%, *aeg* = 21.7%, χ^2^ = 20.7, df = 1, *p*<0.0001; Percentages combine over geographic populations in Table 2). *Aedes albopictus* from Indian River/St. Lucie Co., FL had higher transmission than other geographic locations (Fig 3). *Ae*. *aegypti* from Monroe Co. had significantly higher transmission than from Manatee Co. (χ^2^ = 15.0, df = 1, *p* = 0.0001) and Indian River/St. Lucie Co., FL (χ^2^ = 8.0, df = 1, *p* = 0.0047) (Fig 4). Origin of *Ae*. *albopictus* affected transmission of IOC five to twelve days following ingestion of infected blood (Table 1). *Aedes albopictus* from Indian River/St. Lucie Co. and Manatee Co. had higher transmission than from Alachua Co., FL (Fig 5).

**Fig 6:**
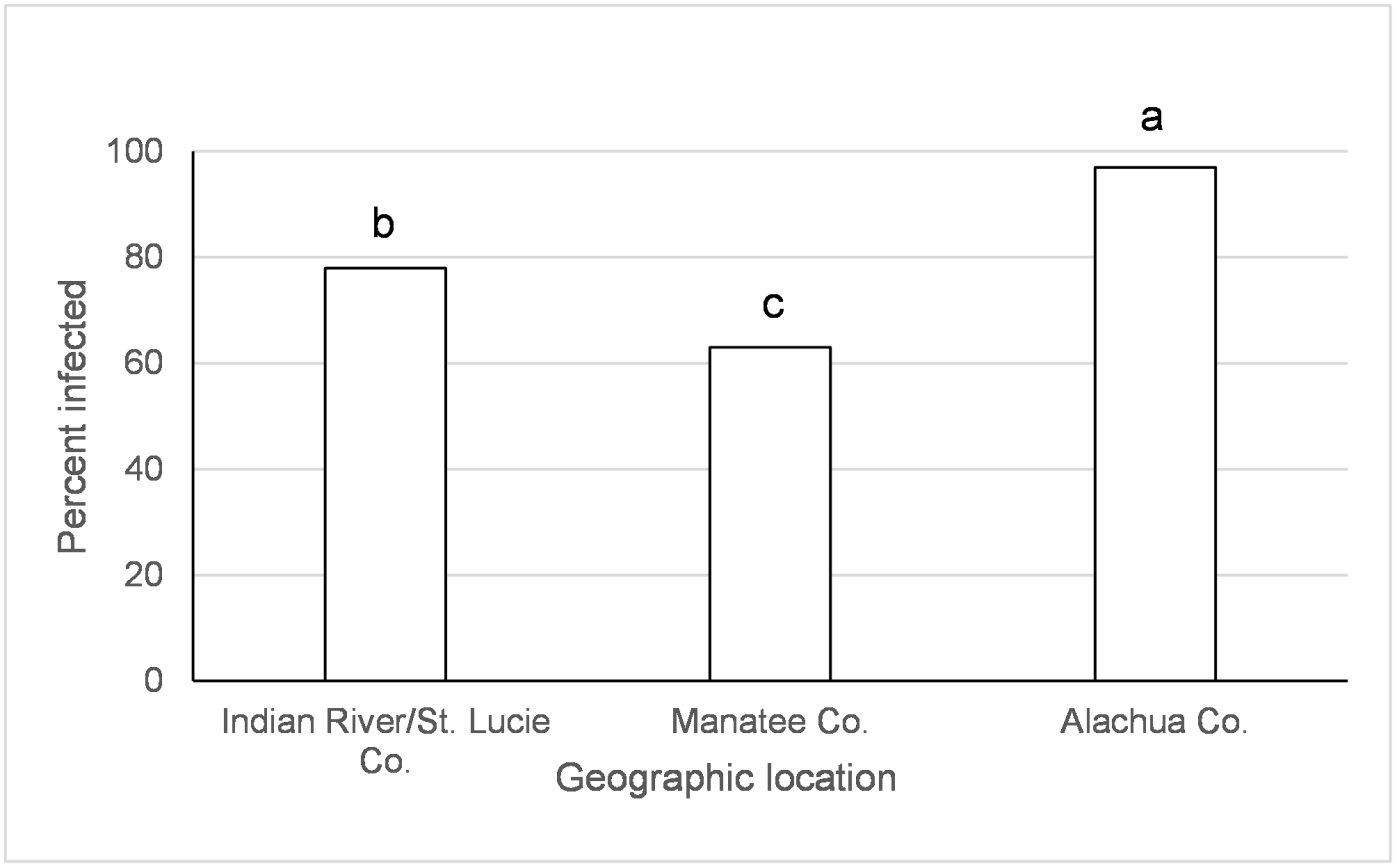
Disseminated infection of the Asian lineage of chikungunya virus in *Ae*. *albopictus*. Values associated with different letters show significant differences after correcting for multiple comparisons using the sequential Bonferroni method. Values average over the time treatment factor.

There was an effect of species, origin of *Ae*. *albopictus* population, and time since exposure on disseminated infection of AC (Table 3). Higher rates of disseminated infection were observed in *Ae*. *aegypti* (80.9%) than *Ae*. *albopictus* (71.2%) (χ^2^ = 4.3, df = 1, *p* = 0.03; Percentages combine over geographic populations and days post infection in Table 4). Disseminated infections increased from day 2 (55.3%) to day 5 (96.2%), then subsequently decreased again on day 12 (82.8%) following exposure. Disseminated infection was significantly different for each of the three time points measured (All χ^2^>21.2 and *p*<0.0001). *Aedes albopictus* from Alachua Co., FL had higher disseminated infection than other locations, followed by Indian River/St. Lucie Co. and Manatee Co. (Fig 6).

**Table 3:** Treatment effects on the Asian lineage of chikungunya virus (British Virgin Islands) disseminated infection (leg infection) and transmission. *Aedes aegypti* = *aeg* and *Ae*. *albopictus* = *albo*.

Disseminated infection		
**Factor**	***χ***^***2***^	*p*
**Species**	4.3	0.036
**Location**	*aeg* = 2.4 *albo* = 14.6	*aeg* = 0.65 *albo* = 0.0007
**Time**	90.6	<0.0001
**Species x Time**	0.84	0.65
**Transmission**		
**Factor**		
**Day 2**	***χ***^***2***^	*p*
**Species**	0.85	0.35
**Location**	*aeg* = 9.79 *albo* = 0.03	*aeg* = 0.04 *albo* = 0.86
**Days 5 and 12**	***χ***^***2***^	*p*
**Species**	0.23	0.62
**Location**	*aeg* = 9.79 *albo* = 1.02	*aeg* = 0.04 *albo* = 0.31
**Time**	32.91	<0.0001
**Species x Time**	7.84	0.0051

**Table 4:** Asian lineage (British Virgin Islands) of chikungunya virus disseminated infection and transmission for *Aedes aegypti* and *Ae*. *albopictus* from different geographic regions of Florida and the Dominican Republic. *Aedes aegypti* = *aeg* and *Ae*. *albopictus* = *albo*.

Geographic region^a^	Mosquito species	Day post infection	% disseminated infection (no. of mosquitoes)^b^	% transmission (no. of mosquitoes)^c^
**Indian River/St. Lucie Co.**	*aeg*	2	64.8 (54)	20.0 (15)
		5	97.9 (48)	75.6 (41)
		12	82.7 (52)	40.5 (42)
	*albo*	2	59.3 (91)	29.6 (27)
		5	96.8 (32)	69.2 (13)
		12	80.6 (62)	25.0 (36)
**Monroe Co.**	*aeg*	2	46.6 (30)	56.3 (16)
		5	94.3 (35)	69.2 (26)
		12	86.9 (46)	60.6 (33)
**Manatee Co.**	*aeg*	2	70.0 (30)	12.5 (8)
		5	95.3 (43)	96.3 (27)
		12	72.5 (40)	37.5 (32)
	*albo*	2	16.6 (30)	33.3 (3)
		5	86.9 (23)	100 (7)
		12	85.7 (14)	33.3 (12)
**Alachua Co.**	*albo*	2	ND	ND
		5	100 (12)	83.3 (12)
		12	94.4 (18)	18.8 (16)
**Dominican Republic**	*aeg*	2	53.6 (28)	6.3 (16)
		5	98.1 (52)	31.1 (45)
		12	81.4 (59)	46.3 (41)
**Laboratory colony (Orlando)**	*aeg*	2	61.9 (21)	37.5 (8)
		5	100 (15)	20.0 (5)
		12	94.4 (18)	0 (9)

^a^Geographic region of mosquito populations collected in Florida and the Dominican Republic.

^b^Disseminated infection corresponds to the percent of infected legs from the total number engorged with blood. A total of 853 mosquitoes were tested.

^c^Transmission corresponds to the percent of saliva-infected mosquitoes from the total number of mosquitoes with infected legs. A total of 490 mosquitoes were tested.

ND, not determined.

**Fig 7:**
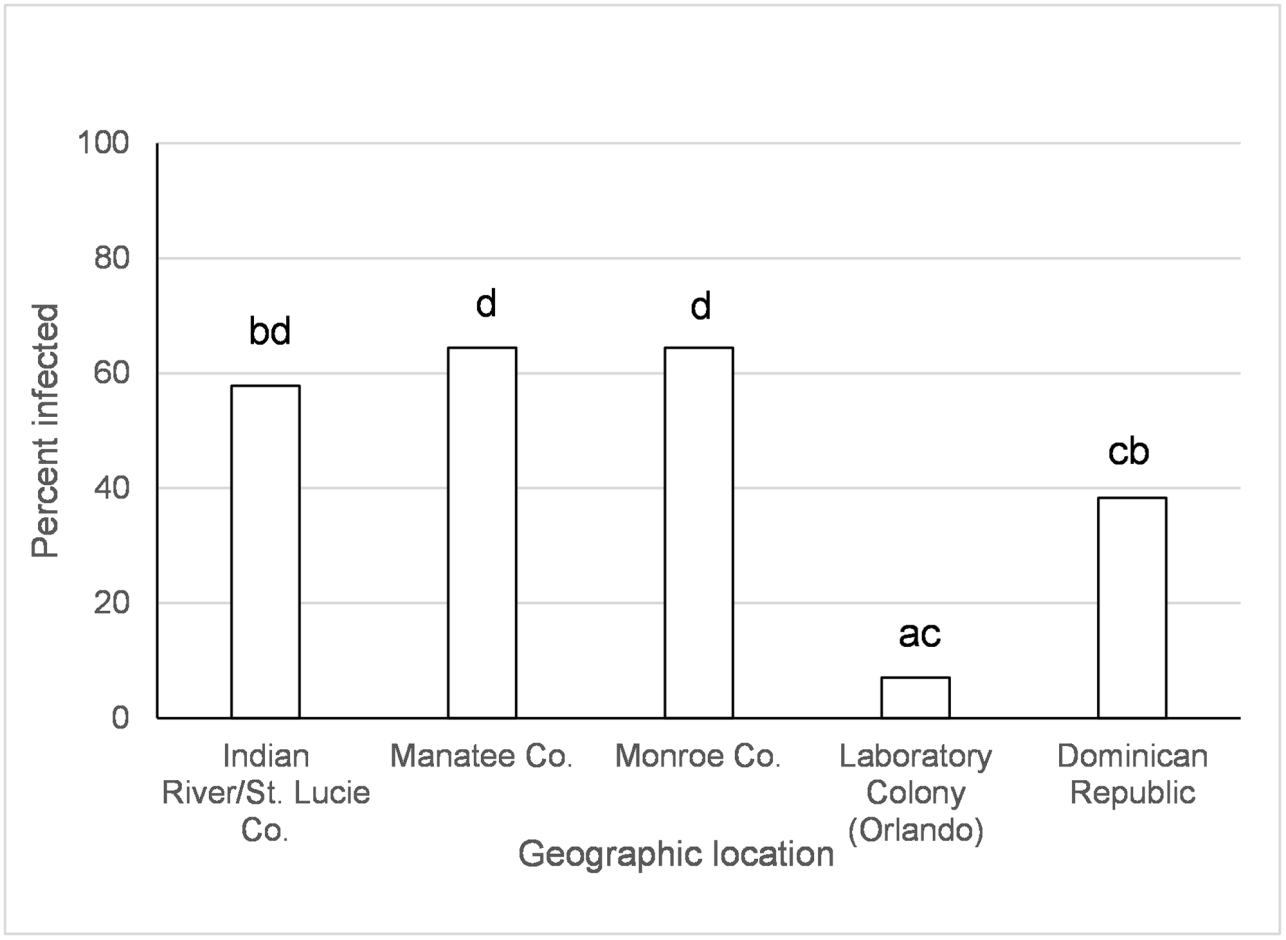
Transmission (days 5 and 12) of the Asian lineage of chikungunya virus in *Ae*. *aegypti*. Values associated with different letters show significant differences after correcting for multiple comparisons using the sequential Bonferroni method. Values average over the time treatment factor.

Origin of *Ae*. *aegypti* population significantly affected transmission of AC two days following ingestion of infected blood (Tables 3 and 4). *Aedes aegypti* from Monroe Co., FL had higher transmission rates than those from the Dominican Republic (χ^2^ = 6.71, *p* = 0.009). However, after correcting alpha for multiple comparisons this difference was only marginally significant. Geographic origin of *Ae*. *aegypti*, time, and the species x time since exposure interaction significantly affected transmission of AC five to twelve days following ingestion of infected blood (Table 3). *Aedes aegypti* from the Dominican Republic and the laboratory colony (Orlando) had similar or lower transmission rates than from all other locations in Florida (Fig 7). Transmission rates decreased during the infection between days five and twelve, but this effect differed between *Ae*. *aegypti* and *Ae*. *albopictus*. *Aedes aegypti* transmission was higher than *Ae*. *albopictus* at twelve days, but not five days, following ingestion of AC infected blood (Day 5 *aeg* vs. *albo*, χ^2^ = 3.76, df = 1, *p* = 0.05; Day 12 *aeg* vs. *albo*, χ^2^ = 6.22, df = 1, *p* = 0.01).

**Fig 8:**
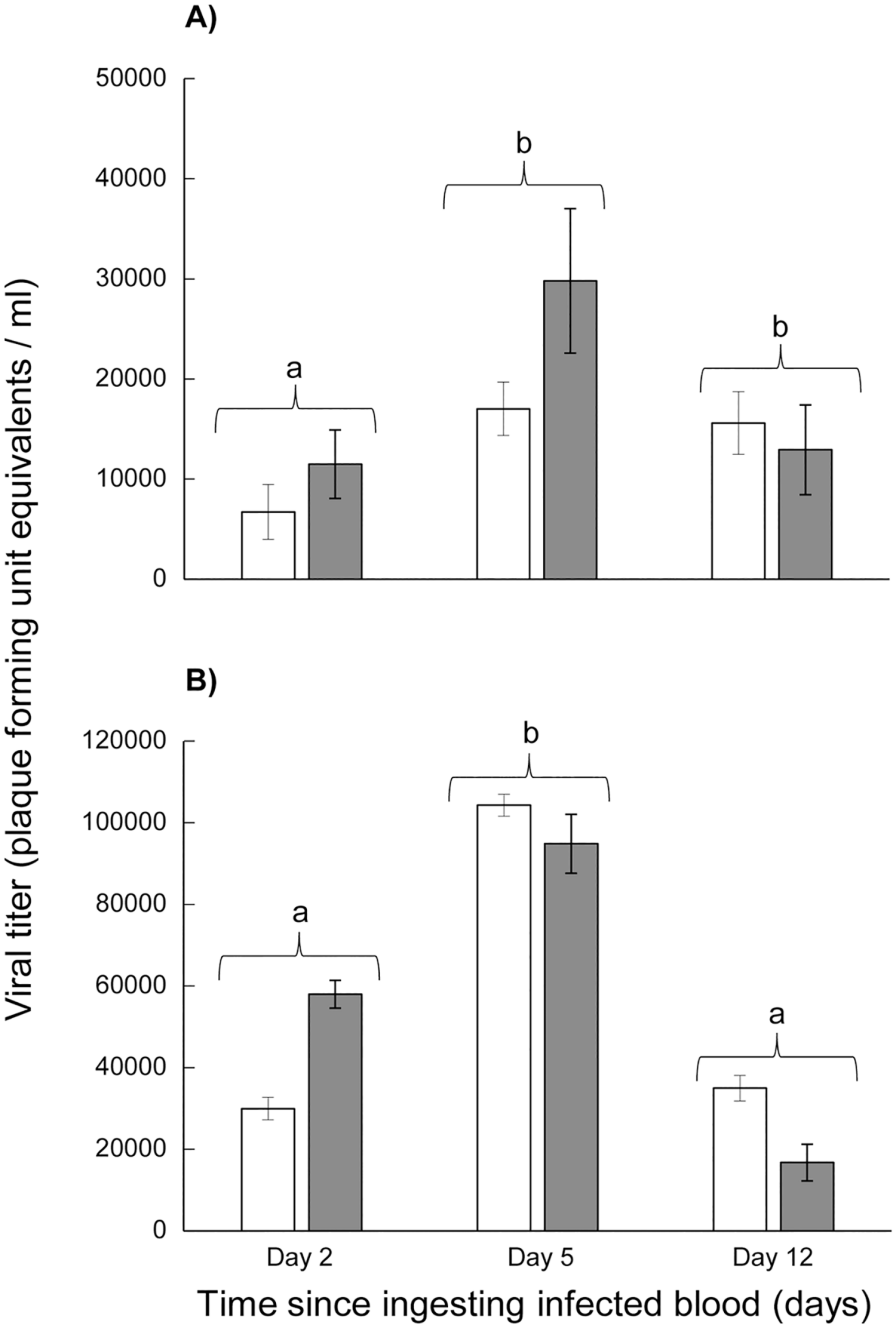
(A) Viral titer of the Indian Ocean lineage of chikungunya virus in *Ae*. *aegypti* legs (white symbol) and *Ae*. *albopictus* legs (grey symbol). Values average over location treatment factor. (B) Viral titer of the Asian lineage of chikungunya virus in *Ae*. *aegypti* legs (white symbol) and *Ae*. *albopictus* legs (grey symbol). Values average over location treatment factor.

There were significant temporal differences in the leg titer equivalents of individuals following exposure to both emergent CHIKV lineages (Table 5). For IOC, viral titer equivalents were significantly higher on days 5 and 12 than day 2 (Fig 8A). For AC, viral titer equivalents were significantly higher on day 5 than days 2 and 12 (Fig 8B). There were no significant treatment effects of species, location, or species by time interaction on leg titer equivalents for either AC or IOC (Table 5). Our ability to detect temporal patterns in saliva viral titer equivalents was limited because we used two different analyses for day two versus days five and twelve. There were no significant differences in viral titer in saliva from the species, location, time, or species by time interaction for either AC or IOC (Table 5). The one exception was that there was a location effect for *Ae*. *albopictus* so that individuals originating from Alachua Co. FL had significantly higher IOC viral load in the saliva than individuals from Indian River/St. Lucie Co. and Manatee Co. FL (Table 5).

**Table 5:** Treatment effects on replication kinetics of Indian Ocean and Asian lineages of chikungunya virus in legs and saliva of *Aedes aegypti* and *Aedes albopictus*. *Aedes aegypti* = *aeg* and *Ae*. *albopictus* = *albo*.

Indian Ocean lineage of chikungunya virus		
**Leg viral titer**		
**Factor**	**F**	*p*
**Species**	1.43	0.23
**Location**	*aeg* = 1.4 *albo* = 0.64	*aeg* = 0.24 *albo* = 0.52
**Time**	5.93	0.0028
**Species x Time**	1.60	0.20
**Saliva viral titer**		
**Factor**	**F**	*p*
**Day 2**		
**Species**	0.97	0.32
**Location**	^ǂ^*aeg* = ND *albo* = 16.58	^ǂ^*aeg* = ND *albo*<0.0001
**Days 5 and 12**	**F**	*p*
**Species**	0.16	0.69
**Location**	*aeg* = 0.95 *albo* = 0.21	*aeg* = 0.41 *albo* = 0.81
**Time**	1.17	0.28
**Species x Time**	0.12	0.72
**Asian lineage of chikungunya virus**		
**Leg viral titer**		
**Factor**	**F**	*p*
**Species**	<0.0	0.99
**Location**	*aeg* = 1.6 *albo* = 0.16	*aeg* = 0.17 *albo* = 0.85
**Time**	25.50	<0.0001
**Species x Time**	2.12	0.12
**Saliva viral titer**		
**Factor**	**F**	*p*
**Day 2**		
**Species**	0.90	0.35
**Location**	*aeg* = 0.89 ^ǂ^*albo* = ND	*aeg* = 0.50 ^ǂ^*albo* = ND
**Days 5 and 12**	**F**	*p*
**Species**	0.46	0.49
**Location**	*aeg* = 0.95 albo = 0.41	*aeg* = 0.43 *albo* = 0.66
**Time**	0.46	0.49
**Species x Time**	0.46	0.49

^ǂ^Analyses were not done (ND) when some treatments had only one sample.

## Discussion

Preliminary studies using *Ae*. *aegypti* mosquitoes from St. Lucie Co., FL and AC identified baseline infection and disseminated infection rates at constant 25°C and 30°C. We observed that *Ae*. *aegypti* had a low susceptibility to infection for AC, but a relatively permissive midgut escape barrier after ingesting a low dose of CHIKV infected blood. A midgut infection barrier refers to the inhibition of ingested arboviruses from entering or replicating in midgut cells. A midgut escape barrier refers to the inhibition of arboviruses from spreading beyond the basal lamina of the midgut cells to the hemocoel. The low infection rates were attributed to a relatively low dose of CHIKV in blood meals. Lack of significant differences among dissemination and transmission rates is most likely attributable to low sample sizes. Our main study demonstrated that the midgut infection barriers can be surpassed by high virus titers [47].

All populations of *Ae*. *aegypti* and *Ae*. *albopictus* mosquitoes displayed susceptibility to infection and transmission for the two emergent lineages of CHIKV at high titers. Viral dissemination to the hemocoel for *Ae*. *aegypti* and *Ae*. *albopictus* mosquitoes was rapid and co-occurred with infection of the saliva, with substantial transmission occurring by day 2 dpi (Tables 2 and 4). This observation has important implications for CHIKV epidemiology because both *Ae*. *aegypti* and *Ae*. *albopictus* exhibit gonotrophic discordance whereby mosquitoes will blood feed more than once in a single gonotrophic cycle [48–51], allowing for the possibility of transmission during each feeding event. Florida vectors are highly competent, especially given the short extrinsic incubation period of CHIKV [44] which strongly contributes to vectorial capacity as an exponential function [52]. Viral disseminated infection indicated rapid propagation in the midgut and spread to other mosquito tissues, with rates being higher for IOC than AC. Viral dissemination within *Ae*. *aegypti* and *Ae*. *albopictus* occurred for most individuals following five days of extrinsic incubation, suggesting a lack of substantial midgut escape barriers for IOC and AC [32]. For IOC, *Ae*. *albopictus* had higher disseminated infection than *Ae*. *aegypti* in most instances, suggesting that *Ae*. *albopictus* is more permissive to infection by this strain than *Ae*. *aegypti*. Similarly, *Ae*. *albopictus* had more efficient transmission of the IOC lineage sooner after ingesting CHIKV infected blood than *Ae*. *aegypti*. Specifically, *Ae*. *albopictus* had a 44% greater proportion of transmission than *Ae*. *aegypti*. However, later in the infection process *Ae*. *aegypti* and *Ae*. *albopictus* had similar transmission. These observations are consistent with other studies showing more efficient viral dissemination for IOC into mosquito secondary organs and transmission in *Ae*. *albopictus* than *Ae*. *aegypti* [27, 32, 53]. We can infer from our observations that the duration of the extrinsic incubation period of IOC is shorter in *Ae*. *albopictus* than *Ae*. *aegypti* [43]. Differences in vector competence between *Ae*. *albopictus* and *Ae*. *aegypti* contribute as a linear function, and so relatively weakly, to vectorial capacity whereas changes in the extrinsic incubation period contribute as an exponential function and thus more strongly [52]. A short incubation period in *Ae*. *albopictus* probably contributed to its role as the vector in the chikungunya outbreaks in the Indian Ocean in 2005–2007. Other contributing factors to this outbreak include an increased infectivity of *Ae*. *albopictus* to this strain by 100-fold and that *Ae*. *aegypti* was relatively rarer and non-anthropophilic on Reunion Island.

Tsetsarkin et al. [21] tested the hypothesis, using viral infectious clones of CHIKV, that a mutation in the envelope protein gene (E1-A226V of IOC) influenced viral fitness for different vector species. Their study demonstrated that the E1-A226V mutation was directly responsible for increased infectivity and more efficient viral dissemination into mosquito secondary organs and transmission for *Ae*. *albopictus* compared to *Ae*. *aegypti* [21]. This adaptive mutation has been selected for on multiple independent occasions, evidence for convergent evolution and the ability of IOC to adapt locally to vectors [54].

Viral dissemination, but not transmission, of AC were lower than IOC two days following ingestion of infectious blood by both *Ae*. *aegypti* and *Ae*. *albopictus*. However, transmission was similar or higher for both mosquito species after five days of extrinsic incubation to AC than to IOC (Tables 2 and 4). Viral dissemination and transmission was higher in *Ae*. *aegypti* than *Ae*. *albopictus* which is consistent with other studies comparing the vector competence of these two species for the Asian lineage of CHIKV [21, 27, 32]. Although viral dissemination rates of AC and IOC were high for most mosquito populations, transmission was lower, suggesting substantial salivary gland infection or escape barriers [32]. Viral dissemination and transmission of AC decreased from 5 to 12 days of extrinsic incubation, suggesting that transmission risk declines with length of infection. Interestingly, the decline in transmission of AC was less in *Ae*. *aegypti* than *Ae*. *albopictus*, suggesting that older *Ae*. *aegypti* females are relatively more competent vectors than similar aged *Ae*. *albopictus* females. Older mosquitoes represent a greater epidemiological threat, because they are more likely to have ingested a virus-infected blood meal and completed the extrinsic incubation period and are more likely capable of transmitting the virus during these advanced stages of infection [55]. Our observations that transmission efficiency declines with length of infection are likely to mitigate age-enhanced transmission potential. Indices of risk of transmission, such as vectorial capacity, do not consider that different species or genotypes of pathogens are affected differently by time since infection, irrespective of daily vector survivorship. Existing models of vector-borne arboviruses could be used to determine whether the pattern of transmission is altered by the addition of time-dependent transmission efficiency during the lifespan of the vector, or by species-specific variation in EIP, transmission or mosquito life history traits. Preliminary studies using a model to investigate the likelihood of CHIKV epidemics in FL after introductions showed differences in the outcome when species-specific variation in vector competence for CHIKV, mosquito mortality and human biting frequency were considered. Ongoing research is investigating effects of the variation in EIP observed in this study and will be published elsewhere. One plausible mechanism that would account for decline in transmission efficiency with length of infection is virus modulation of the infection by the mosquito [56]. This hypothesis predicts that reduced viral titer in mosquito tissues would be observed on day 12 compared to day 5 because of the observed decline in transmission at the later time point. We would thus expect to see changes in leg viral titers in association with changes in saliva infection over time. The observed reduction in leg viral titer, in association with reduction in transmission, for AC supports this hypothesis. Further support comes from the observation that saliva infection did not decline over time in IOC, and neither did leg viral titer. Salazar et al. [57] showed that after ingestion of dengue-2 virus, peak virus titer in *Ae*. *aegypti* was observed after 7–10 days of extrinsic incubation followed by a steady decline later during infection. Although the molecular mechanism responsible for reduced virus load was unclear, the authors suggested several potential means including physiologically compromised epithelial cells, post-transcriptional or post-translational repression, or an antiviral response [57]. Similar observations were made with *Ae*. *aegypti* infected with dengue-2 by Sánchez-Vargas et al. [58] in which they showed that dengue infection and viral titer in *Ae*. *aegypti* were modulated by the RNAi defense system. Additional experiments are needed to identify the mechanism(s) responsible for modulation of infection observed in the current study.

Large scale geographic variation in vector competence of *Ae*. *aegypti* and *Ae*. *albopictus* have been observed among lineages of CHIKV [32, 59] as well as for other arboviruses (Zika, yellow fever, and dengue-2 viruses) [60–64]. In the current study, we have identified variation in vector competence on a smaller spatial scale than previously recognized. Regional differences in mosquito-virus interactions, especially as they might relate to the EIP, may have important implications for risk of disease transmission. However, the geographic variation wasn’t consistent between CHIKV lineages and mosquito species and the differences were often relatively small. *Aedes aegypti* from Manatee Co., Florida and Dominican Republic had lower or similar viral dissemination of IOC from one or more other locations in Florida (Fig 2). Along the same lines, transmission was observed to be lower for *Ae*. *aegypti* from Manatee Co. and from Indian River/St. Lucie Co. than Monroe Co (Fig 4). A study on the phylogeography of *Ae*. *aegypti* in Florida did not find strong genetic differentiation among Florida populations of *Ae*. *aegypti* from East and West coasts, but there was some evidence of genetic isolation of Florida Keys *Ae*. *aegypti* from mainland [37] which may, in part, explain our observation. Similar studies characterizing genetic differentiation among Florida populations of *Ae*. *albopictus* have not yet been published. Studies of *Ae*. *aegypti* from single nucleotide polymorphisms and sequenced nuclear genes have demonstrated differences in populations from the Caribbean compared to mainland U.S. [65], which is consistent with large scale difference in quantitative genetics of vector competence [66]. For *Ae*. *albopictus*, although IOC disseminated infection was high and homogenous between collection sites, transmission rate was much lower and varied by origin, suggesting distinct barriers to transmission that may be operating at small geographic scales (Figs 3 and 5). Specifically, *Ae*. *albopictus* from Alachua Co. had lower IOC transmission than individuals originating from either Manatee Co. or Indian River/St. Lucie Co., FL, with the latter location resulting in the higher transmission potential. In contrast, *Ae*. *albopictus* from Alachua Co. had higher BVIC viral dissemination than individuals from Manatee or Indian River/St. Lucie Counties (Fig 6). However, this effect did not correspond to similar changes in transmission. *Aedes aegypti* from the Dominican Republic and the laboratory strain had lower transmission potential than recent colonies from Florida (Fig 7). Taken together, these observations suggest complex interactions between mosquito and CHIKV genotypes. *Aedes aegypti* originating from the Dominican Republic had viral dissemination and transmission potential rates for IOC and AC that were lower than Florida vectors (Figs 2 and 7). The fact that a large outbreak of CHIKV occurred in the Dominican Republic indicates that these lower rates are still sufficiently high to sustain transmission in nature and suggests that other factors largely contribute to transmission [60, 67, 68], such as biting rates of humans by the vectors. To our knowledge there are no entomological surveys in Florida during the 2014 outbreak that would incriminate potential mosquito species as being infected with CHIKV. However, local infections in 2014 occurred in the ranges of both potential vector species in Florida.

Our experiments demonstrated variation in dissemination and transmission among mosquito populations and virus strains, however, in some instances sample sizes were low limiting our ability to detect differences. Logistic constraints limited the number of time points we were able to sample, affecting precision in estimating minimum EIP and viral dynamics in the mosquitoes. With these constraints, however, we were still able to show geographic variation in vector competence. As similar variation may occur in other mosquito species, our results highlight the need for detailed investigations of vector competence across species and populations in different regions. Although geographic differences in the vector competence described in the current study may modulate local risks of infection and transmission, other components of vectorial capacity, such as vector survivorship and human biting rate, are likely to be more important determinants of the potentials for epidemic or endemic transmission [52]. Additionally, biting rate is probably enhanced by the abundance of breeding sites associated with water storage and irregular trash collection, suggesting that source reduction may play an important role in reducing transmission risk.
